# Information technology and pre-hospital Emergency centers and the emergency room in the hospital 

**Published:** 2019-08

**Authors:** Davoud Kamali, Yousef Babakhani Omid

**Affiliations:** ^*a*^M.Sc. of Medical Informatics Technology, Biomedical Engineering Department, Amirkabir University of Technology, Tehran, Iran.; ^*b*^M.Sc. student of Health Technology Assessment, Kerman, Iran.

## Abstract

Car accidents and traffic accidents incidents represents one of the greatest challenges to a community’s emergency response system. Rescuers, ﬁeld medical personnel, and regional emergency departments and hospitals must often provide care to large numbers of casualties in a setting of limited resources, inadequate communication, misinformation, damaged infrastructure, and great personal risk. Emergency care providers and incident managers attempt to procure and coordinate resources and personnel, often with inaccurate data regarding the true nature of the incident, needs, and ongoing response. New technologies in communications, the Internet, computer miniaturization, and advanced ‘‘smart devices’’ have the potential to vastly improve the emergency medical response to such car accident disasters. In particular, next generation wireless Internet and repositioning technologies may have the greatest impact on improving communications, information management, and overall disaster response and emergency medical care. One consistent challenge for disaster response is communication and information management. Effective response requires a moment-to-moment ‘‘situational analysis’’ and real-time information to assess needs and available resources that can change suddenly and unexpectedly. A critical ‘‘sequential interdependence’’ exists: accurate information from the ﬁeld about the incident, casualties, medical needs, triage, and treatment impacts the utilization and preparedness of community resources such as ambulances, EDs, hospitals, and intensive care units. Similarly, information on available and accessible hospital, ED, and ambulance resources alters the management and disposition of victims at the scene. The major problem at emergency room (ER) is access to specialist when they are elsewhere in the hospital. The nurse pages the doctor about the emergency. The specialists do not respond because they are busy with other patients in other place or something else. Radio Frequency Identification [RFID] can help us to track doctors, nurses or patients to render better health services to cure patients. This study reviewed the literature on the information technology applications in prehospital emergency center and emergency room in hospital. The literature search was conducted with the help of library, data bank, and also searches engines available at Google, and conference proceedings.

**Keywords:**

Health Information Technology, Emergency center, Emergency room

